# Detection of Hepatitis E Virus Antibodies in Dogs in the United Kingdom

**DOI:** 10.1371/journal.pone.0128703

**Published:** 2015-06-15

**Authors:** Aoife McElroy, Rintaro Hiraide, Nick Bexfield, Hamid Jalal, Joe Brownlie, Ian Goodfellow, Sarah L Caddy

**Affiliations:** 1 Division of Virology, Department of Pathology, University of Cambridge, Addenbrookes Hospital, Hills Road, Cambridge, CB2 2QQ, United Kingdom; 2 School of Veterinary Medicine and Science, University of Nottingham, Sutton Bonington Campus, School Lane, Sutton Bonington, Leicestershire, LE12 5RD, United Kingdom; 3 Public Health England, Public Health Laboratory Cambridge, Addenbrookes Hospital, Hills Road, Cambridge, CB2 2QQ, United Kingdom; 4 Department of Pathology and Pathogen Biology, The Royal Veterinary College, Hawkshead Lane, North Mymms, Hatfield, Hertfordshire, AL9 7TA, United Kingdom; 5 Section of Virology, Faculty of Medicine, Imperial College London, St. Mary's Campus, Norfolk Place, London, W2 1NY, United Kingdom; Centers for Disease Control and Prevention, UNITED STATES

## Abstract

Hepatitis E virus (HEV) genotypes 3 and 4 are zoonotic pathogens, with pigs predominantly implicated in disease transmission. The rapid rise in human cases in developed countries over the past decade indicates a change in epidemiology of HEV, and it has been suggested that additional animal species may be involved in transmission of infection. Multiple studies have identified contact with dogs as a risk factor for HEV infection in industrialised nations, and a low seroprevalence to HEV has previously been reported in dogs in low-income countries. In this study we aimed to evaluate the possibility that dogs are susceptible to HEV, and determine the frequency with which this occurs. Serum samples from UK dogs with and without hepatitis were screened for HEV-specific antibodies, and canine liver and stool samples were analysed by qPCR for the presence of HEV RNA. We describe evidence to show HEV infection occurs at low levels in dogs in the UK, but the strain of origin is undetermined. The low seroprevalence level of HEV in dogs implies the risk of zoonotic disease transmission is likely to be limited, but further investigations will be required to determine if HEV-infected dogs can transmit HEV to man.

## Introduction

Hepatitis E Virus (HEV) is a major cause of acute viral hepatitis in developing countries [1] and has recently emerged as the most common cause of acute hepatitis in the UK [2]. HEV is a icosahedral, single stranded, positive sense RNA virus with a genome of approximately 7.2kb, first identified as a cause of disease in 1983 [3]. There are four HEV genotypes; genotypes 1 and 2 are endemic in humans in developing regions, whilst genotypes 3 and 4 are zoonotic agents associated with sporadic outbreaks of HEV worldwide [4].

The first animal strain of HEV was identified in pigs in the US [5]. Porcine HEV strains are antigenically and genetically related to human strains of HEV, and experimental evidence has shown that cross species infection can occur between humans and pigs [6]. Multiple studies have sought to determine whether other animal species could be additional zoonotic sources of HEV infection to man. Anti-HEV antibodies have been identified in a range of farmed animals, including cattle, goats and chickens [7,8], but their role in transmission of disease to man is unclear.

Pet dogs have been implicated in HEV disease transmission in several previous reports. There are an estimated 9.4 million dogs in the UK [9], hence identification of dogs as a possible zoonotic reservoir could have serious public health consequences. Anti-HEV antibodies have been identified in dogs in developing countries where HEV is endemic in humans, including China, India and Brazil [7,10–14]. Two studies have previously investigated the seroprevalence of HEV in dogs from regions with sporadic HEV cases in humans; no positive samples were identified in Japan, and 2/212 positive dogs were identified in the US [15,16]. Despite this very low seroprevalence, an epidemiological link between HEV infection and dogs has still been implied in industralised nations. Periodic contact with dogs was reported in 74% (14/19) of cases of indigenously acquired infection in a Dutch study [17], and owning pets was reported by 60% (17/28) of patients with indigenous HEV infection from the UK [18].

Confirmation of the ability of HEV to infect dogs requires identification of HEV RNA within canine samples. Previous studies in Asia have examined canine stool and serum samples for HEV RNA, but no positive cases have been identified, despite using primers that target highly conserved regions of the HEV genome [12,13,15,16]. Similarily, no HEV RNA was detected in a Dutch study examining canine liver samples collected prior to 2005 [19].

Given the rise in human HEV cases with a suspected zoonotic origin in the past decade [20], it is possible that previous attempts to identify HEV RNA in canine samples have produced negative results due to very low prevalence levels. Therefore, this study aimed to investigate the potential for dogs to be infected with HEV using stool, serum and liver samples collected from dogs in the UK over the past five years. As the structure of the HEV capsid enables self-assembly into virus-like particles (VLPs) [21–23], we generated HEV VLPs to screen samples for the presence of anti-HEV antibodies. We also tested canine samples for the presence of HEV RNA using qRT-PCR.

## Material and Methods

### Samples

Serum samples were collected from three groups of pet dogs in the UK. Samples in the first two groups were collected in 2012/2013. The first of these were patients presenting to the Royal Veterinary College, University of London, and the second were healthy blood donors for the UK Pet Blood Bank, Loughborough. In both groups, blood samples consisted of that remaining after routine diagnostic testing. All samples were collected with written and informed owner consent. The third group of dogs all had a histological diagnosis of hepatitis, recruited to previous studies [24,25]. All serum samples were collected and stored at -20°C prior to screening in ELISAs for their ability to detect HEV virus-like particles (VLPs).

Stool samples were collected from dogs admitted to five participating veterinary clinics and an animal shelter across the south and east of England, as part of a previous study [26]. The presence or absence of liver disease in any cases was not reported. Stool samples were also collected from healthy dogs owned by veterinary staff at each clinic, as well as from dogs at participating boarding kennels. All stool samples were stored at -20°C until and during transportation to the laboratory, whereafter they were stored at -80°C prior to nucleic acid extraction.

Canine liver samples were obtained from dogs with a histological diagnosis of hepatitis following liver biopsy. All samples were collected with written and informed owner consent and with the approval from the Institutional Ethics and Welfare Committee. As previously described [24], liver tissue was immediately frozen at -80°C, or collected into RNAlater (Life Technologies Ltd), then stored at 4°C for up to 24h and frozen at -80°C.

### Virus-like particle (VLP) production

HEV VLPs were produced following generation of recombinant baculoviruses expressing the HEV ORF2 gene using the flash BAC baculovirus expression system (Oxford Expression Technologies). DNA encoding the 112–608 amino acids of the HEV genotype 3 ORF2 gene was amplified by PCR from an infectious cDNA clone of HEV strain Kernow (clone p6; GenBank accession no. JQ679013, a kind gift from S. Emerson). Recombinant baculoviruses were propagated in Sf9 cells, then VLP expression was achieved in Hi-5 insect cells. VLPs were purified according to previously published protocols, which have demonstrated the generation of icosahdral particles by EM [23,27]. Purification involved centrifugation for 1h at 10,000xg at 4°C to remove cell debris, then the supernatant was centrifuged at 100,000xg for 8-10h through a 30% sucrose cushion at 4°C. VLPs were further purified by centrifugation for 24h at 110,000xg at 4°C in an isopycnic caesium chloride gradient. A single band was obtained at a density of 1.31g/ml, in agreement with previous reports. VLPs were isolated and concentration analysed by BCA assay and visualized by coomassie staining. GI.1 norovirus and vesivirus 2117 VLPs were generated using a similar method as previously described [26].

### ELISA protocol

ELISAs were performed by coating 96-well polystyrene microtitre plates (Nunc maxisorb, Fisher Scientific) overnight at 37°C with 50ng of HEV VLPs in 0.05 M carbonate/bicarbonate buffer (pH 9.6). 0.05% Tween 20 in phosphate buffered saline (PBS-T) was used to wash the plates three times before blocking with 5% milk-PBS-T for 1h at 37°C. A further three PBS-T washes were carried out, then plates incubated for 3h at 37°C with 1:50 dilution of each serum sample in duplicate. Mouse anti-HEV monoclonal antibody was used as a positive control. After three washes with PBS-T, horseradish peroxidase (HRP)-conjugated anti-dog IgG antibody (Sigma Aldrich) was added to each well and incubated at 37°C for 1h. Three more washes with PBS-T were performed and bound antibody was detected with tetramethylbenidine (TMB, Sigma Aldrich). Reactions were stopped with 1N H_2_SO_4_ and optical density (OD) read at 450 nm (Spectromax iE plate reader, Molecular Devices). The OD450 value for serum samples incubated in wells coated with buffer alone defined the background signal for each sample. Background signal was then subtracted from the OD450 of VLP coated wells to generate the corrected OD450 value. The ELISA cut-off threshold was established as the mean of the OD450 of all buffer coated cells plus 3 standard deviations. Antibody titres for any positive samples were obtained by two-fold serial dilution of samples.

### Nucleic acid extraction and qRT-PCR

Canine stool samples were diluted 10% w/v in phosphate-buffered saline (pH 7.2), and solids removed by centrifugation at 8000 x g for 5 min. Viral nucleic acid was extracted from 140μl clarified stool suspension by the GenElute Mammalian Total RNA Miniprep Kit (Sigma Aldrich) according to the manufacturers’ instructions.

Canine liver samples were homogenized in 500μl of lysis buffer and β-merceptoethanol with 1mm silica beads (BioSpec products) using a reciprocating homogeniser (FastPrep-24, MP Biomedicals). RNA was extracted using the GenElute Mammalian Total RNA RNA Miniprep Kit (Sigma Aldrich) according to the manufacturers’ instructions, then DNAse treated (Roche). RNA was extracted using the same commerical kit from 100μl serum samples.

RNA was transcribed *in-vitro* from an HEV infectious clone [28] and used as a positive control. The full length clone was linearised with MluI, then 250ng was added to 0.1M HEPES (pH 7.5), 32mM MgAcetate, 40 mM dithiothreitol (DTT), 2mM spermidine, 7.5 mM ATP, CTP, GTP, and UTP (each), 2.5μg T7 polymerase, and 80 units of RNaseOUT (Life Techologies) in a 50μl volume. The *in-vitro* transcription reactions were carried out at 37°C for 2h. Afterwards, the reaction mixtures were incubated with 20 units of DNase I (Roche) at 37°C for 30min. The RNA was then purified by ethanol precipitation and resuspended in RNA storage solution (Ambion). The RNA was quantified using spectrophotometry and stored at -20°C until further use.

One step qRT-PCR was performed using primers designed to detect all four genotypes of HEV, targeting a conserved region of HEV ORF3 [29]. qPCR reactions were prepared using 2μl of extracted RNA added to 2x Precision OneStep qRT-PCR MasterMix (PrimerDesign Ltd), with primers and probe at concentrations of 250 and 100 nM, respectively. The thermal cycle protocol used with a ViiA7 qPCR machine (AB Applied Biosystems) was as follows: RT reaction 50C for 30min, 95°C for 15min, then 42 cycles of 95°C, 10sec; 55°C, 25sec; 72°C, 25sec. Samples with any evidence of amplification by qPCR were analysed by gel electrophoresis.

### SDS-PAGE and western blot analysis

HEV VLPs as well as GI.1 norovirus and vesivirus 2117 VLPs included as controls, were separated on a SDS-polyacrylamide gel, then samples were either transferred to polyvinylidene difluoride membranes for western blotting, or stained with Coomassie Blue. Membranes were blocked for 1h at room temperature with 5%-milk-PBS—T, then incubated overnight at 4°C with serum samples diluted 1:500. After washing three times with PBS-T, membranes were incubated for 1h with HRP-conjugated secondary antibodies. After washing with PBS-T, bands were detected using ECL reagents (GE Healthcare).

## Results

### Prevalence of HEV antibodies in canine sera

**Fig 1:**
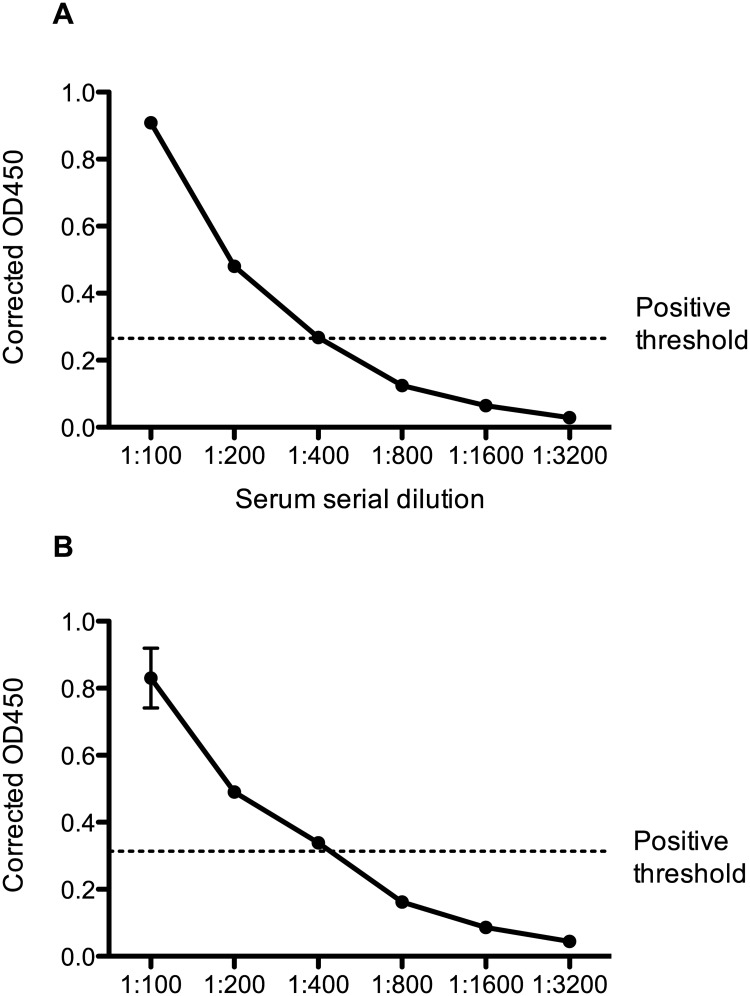
Anti-HEV antibody titres in positive canine serum samples. Positive canine serum samples were prepared in dilutions of 1:100, 1:200, 1:400, 1:800; 1:1600 and 1:3200 and used in an ELISA assay. The corrected OD450 was obtained by subtracting the background signal from the VLP coated well OD450 value. The positive threshold was determined by calculating the mean OD450 of buffer coated wells with the highest serum dilution, plus 3 standard deviations.

A total of 247 canine serum samples were collected and screened by ELISA for anti-HEV antibodies. Of these samples, 92 were from healthy dogs, 34 were from patients with a range of unspecified clinical diseases at a veterinary hospital, and 121 were from dogs with confirmed hepatitis. Two dogs were identified as seropositive, both from the group of healthy animals (2/92, 2.2%). The antibody titre of the two positive samples (designated samples A and B) was determined to be 1:400 by serial dilution of sera as shown in Fig 1. No seropositive cases were identified amongst the dogs with any clinical signs of disease.

**Fig 2:**
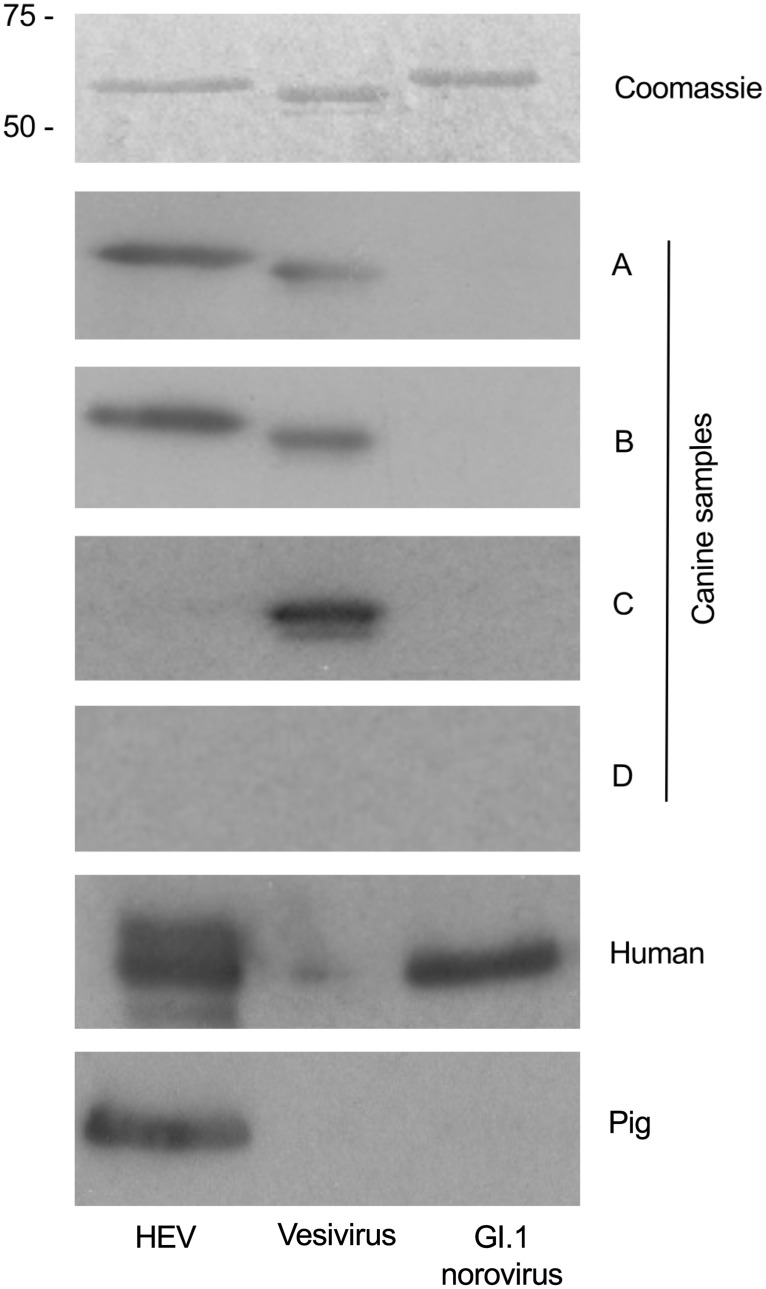
Western blot analysis of serum sample reactivity with HEV, vesivirus 2117 and human norovirus G1.1 VLPs. Three types of VLP were separated by SDS-PAGE. One gel was stained with Coomassie Blue to identify VLP protein at the expected molecular weight. Additional gels were used for western blotting with canine serum samples positive by ELISA for HEV (samples A and B). A pig serum sample (kind gift from S. Emerson) and a human serum sample known to be positive for anti-HEV antibody were used as a positive control for the HEV VLPs. Canine sample C, previously confirmed positive for anti-vesivirus antibody by ELISA, was used as a positive control for the vesivirus VLPs. Canine sample D was used as a negative control for all VLPs.

Western blotting was used as an additional means of confirming samples positive by ELISA. Fig 2 confirms that antibodies present in samples A and B could detect HEV as predicted. These two samples were also seropositive for vesivirus 2117 (a calicivirus related to canine vesivirus), although this is not believed due to be cross-reactivity as demonstrated by inclusion of sample C (positive for vesivirus 2117 only). Canine sample D represents a dog seronegative by ELISA to all VLPs analysed, with the same result demonstrated in the western blot, adding additional support to the conclusion that samples A and B are specific for HEV. Sera from an HEV-seropositive pig and a seropositive human were included as positive controls. Human reactivity to GI.1 norovirus was not unexpected given that approximately 70% human population in the UK are seropositive to this virus [30].

Both samples positive for HEV were collected in August 2013 at separate canine blood collection sessions in the UK. One positive dog was 5 years old and the other was 10 years old. The age of serongeative dogs was known for 97/124 samples, with the average age being 6.7 years (SD 2.19). The two seropositive animals were both of greyhound type breeds. A variety of dogs breeds were included in this study, although larger dogs were overpresented in the healthy cohort due to the population studied (blood donation requires dogs to be heavier than 25kg).

### Prevalance of HEV RNA in canine samples

Optimization of the previously published qRT-PCR protocol for HEV RNA detection [29] was first performed using in-vitro transcribed HEV RNA from an infectious clone. This demonstrated that 100 genome copies of HEV per reaction were reliably detected. Following on from this, a total of 2.5 x 10^7^ genome copies of HEV RNA were spiked into a single canine liver sample, serum sample and a stool sample, to determine the effect that additional material in clinical samples had on assay sensitivity. RNA extraction was conducted according to the protocol described, then 2μl of the extracted RNA was entered into the qRT-PCR reaction, corresponding to 10^6^ copies of HEV RNA per reaction. HEV RNA added directly to lysis buffer in the absence of any canine samples was included as a control. It was demonstrated that HEV RNA could be detected in spiked canine liver and stool samples at levels comparable to the lysis buffer-only control. However, detection of HEV RNA from the spiked serum sample was significantly impaired, with almost a 3 log decrease in the number of copies detected by qPCR. Therefore serum samples would have to contain at least 10^5^ genome copies/μl to be detectable by this assay, which was deemed highly unlikely based an average HEV RNA concentration of 10^5^–10^8^ genomes copies/ml in infected human serum [31]. Hence screening for HEV RNA was conducted for RNA extracted from liver and stool samples only.

A total of 248 canine stool samples were screened for the presence of HEV RNA using the qPCR protocol with primers designed to amplify a highly conserved 71bp fragment of ORF3. Products of any samples showing uncertain results were analysed by gel electrophoresis. A positive control was included on each plate to ensure qPCR had been performed correctly. No HEV positive cases were identified.

Eighty-four canine liver samples were analysed for the presence of HEV RNA using the same qRT-PCR protocol. In all other species studied, HEV induces varying degrees of hepatitis [32–34], hence to optimise the chances of identifying HEV RNA, liver samples from dogs with histologically diagnosed hepatitis were screened in this study. No HEV RNA was detected in any canine liver samples.

## Discussion

This study has shown that of 247 dogs tested from the UK, two (0.8%) were positive for antibodies specific for HEV. This strongly suggests that HEV had replicated within the two positive animals, inducing an immune response. This is the first study investigating the seroprevalence of anti-HEV antibodies in dogs in Europe, and the low canine HEV seroprevalence is similar to the low human seroprevalence in this region. Seroprevalence of HEV in humans in the UK is 13% [35], whereas up to 25% people are seropositive in China [36] where canine HEV seroprevalence can be up to 29% [14]. This also correlates with the negative HEV canine serological study from Japan [15], where human seroprevalence is only 3.4% [37].

An alternative explanation for the two seropositive cases could be the existance of a unique canine-specific HEV strain. All HEV genotypes described to date exist as a single serotype [38], thus serology alone is not able to determine the origin of the HEV strain inducing the immune response. This is exemplified by the recently identified rabbit-specific strains of HEV; these are genetically distinct from human strains, but they share the same serotype as the human genotypes 1–4 [39]. To ascertain whether human, swine or a putative canine HEV strain is inducing an HEV-specific immune response in dogs, identification and characterisation of HEV RNA from canine samples is required.

Multiple studies have previously attempted to identify HEV RNA in canine samples. The majority of these have analysed canine serum [11–13], with no positive cases reported. Experimental infection of dogs with swine HEV has been shown to induce an antibody response, but despite this no HEV RNA was detectable in serum post innoculation [12]. This suggests viraemia is not readily identified in infected dogs, and this coupled with our optimization experiments that failed to reliably detect HEV RNA spiked into serum samples, indicated that analysis of serum samples is not the optimum method for RNA detection in dogs.

In order to increase the chances of identifying HEV RNA in canine samples, alternative clinical samples were deemed necessary. It has been shown that HEV induces hepatitis in a range of species, hence it was assumed that HEV replication in dogs would occur in the liver. Detection of HEV RNA in the liver is possible for up to 1 month post innoculation in experimentally infected pigs, which is twice the duration of viral detection in serum samples [32,40]. It was therefore hypothesised that analysis of canine liver samples would be the most likely strategy to detect virus. Hepatitis is frequently reported in dog populations [41] and yet the aetiology of most cases of canine hepatitis remains unknown despite numerous studies to identify a viral cause [19,24,25]. HEV was included in a PCR screen for a viral cause of canine liver disease in a study of dogs with hepatitis over a decade ago, but no HEV positive cases were identified [19]. The incidence of HEV in man in the UK has increased dramatically over the intervening ten years, and we predicted that HEV might be more frequent in canine hepatitis samples collected more recently. However, despite this reasoning we were not able to detect HEV RNA in the canine liver samples screened from the UK. This is in direct contrast to recent surveys of livers from pigs and wild boar in Europe, where almost 15% samples were HEV positive [42,43].

It is important to note that the primer-probe set used for our qRT-PCR screen targeted a highly conserved region of HEV ORF3. Although originally designed to detect HEV genotypes 1–4 [29], sequence analysis showed that this primer-probe set should also detect the more distantly related HEV strains identified in rabbits and camels. A rabbit HEV strain (GenBank Accession number KJ013414) and a camel HEV strain (KJ496143) had 94–100% sequence identity to the qRT-PCR primer-probe sequence. It was therefore theorised that any novel canine HEV strains should also be detectable using this assay.

A common method of detecting HEV RNA in pigs is from stool samples [44,45], and hence to extend our study we also tested 248 canine stool samples for HEV RNA using qPCR. Again, no positive canine samples were identified which differs from the detection levels in pigs; HEV RNA has been reported in stools of 5–35% pigs across the UK [44,45]. Clearly viral shedding by dogs is substantially lower than in pigs.

Altogether, samples from over 500 dogs have been screened by qPCR or ELISA in this study. However, in spite of being a sizeable cohort in comparison to many canine studies, this number of cases is still significantly fewer than several of the large-scale human studies where over 10,000 human samples were analysed [46–48]. Surveys this large have been required to detect HEV in healthy humans, where prevelance is as low as 1 in 3000 individuals [46]. Studies of this magnitude would be extremely challenging to achieve in canine populations, which means determining accurate prevalence levels of rare viruses is seldom possible.

The zoonotic potential of HEV in dogs was recently assessed by an alternative method in a serosurvey of veterinarians. It was hypothesised that if dogs were a common source of HEV, then humans with high exposure to dogs would be more likely to be seropositive. However it was found that HEV seroprevalence did not increase with occupational exposure to dogs [49]. This is at odds with a serosurvey of swine veterinarians, which found significantly higher levels of seropositivity to HEV in comparison with people not in regular contact with swine [50]. These human prevalence studies indicate that even if it is definitively proven that dogs can become infected with human strains of HEV, they play a much lesser role in disease transmission than pigs.

To summarise, this study has provided evidence that dogs in the UK can become infected with HEV, although strains involved are unknown. The low seroprevalence coupled with the absence of detection of HEV RNA suggests HEV infection is very rare in this cohort of dogs studied. Nonetheless, these preliminary findings warrant further investigations to determine if contact with HEV infected dogs could be a transmission route for HEV to man.
